# The Anticancer Action of a Novel 1,2,4-Triazine Sulfonamide Derivative in Colon Cancer Cells

**DOI:** 10.3390/molecules26072045

**Published:** 2021-04-02

**Authors:** Agnieszka Gornowicz, Anna Szymanowska, Mariusz Mojzych, Robert Czarnomysy, Krzysztof Bielawski, Anna Bielawska

**Affiliations:** 1 Department of Biotechnology, Medical University of Bialystok, 15-222 Bialystok, Poland; anna.szymanowska@umb.edu.pl (A.S.); anna.bielawska@umb.edu.pl (A.B.); 2 Department of Chemistry, Siedlce University of Natural Sciences and Humanities, 08-110 Siedlce, Poland; mmojzych@yahoo.com; 3 Department of Synthesis and Technology of Drugs, Medical University of Bialystok, 15-222 Bialystok, Poland; robert.czarnomysy@umb.edu.pl (R.C.); kbiel@umb.edu.pl (K.B.)

**Keywords:** colorectal cancer, apoptosis, cell signaling, anticancer agents, pyrazolo[4,3-*e*]tetrazolo[1,5-*b*][1,2,4]triazine, 5-fluorouracil

## Abstract

Cancer therapy is one of the most important challenges of modern medical and chemical sciences. Among the many methods of combating cancer, chemotherapy plays a special role. Imperfect modern chemotherapy justifies continuing the search for new, more effective, and safe drugs. Sulfonamides are the classic group of chemotherapeutic drugs with a broad spectrum of pharmacological activity. Recent literature reports show that sulfonamide derivatives have anti-tumor activity in vitro and in vivo. The aim of the study was to synthesize a novel 1,2,4-triazine sulfonamide derivative and check its anticancer potential in DLD-1 and HT-29 colon cancer cells. The biological studies included MTT assay, DNA biosynthesis, cell cycle analysis, Annexin V binding assay, ethidium bromide/acridine orange staining, and caspase-8, -9, and -3/7 activity. The concentrations of important molecules (sICAM-1, mTOR, Beclin-1, cathepsin B) involved in the pathogenesis and poor prognosis of colorectal cancer were also evaluated by ELISA. We demonstrated that the novel compound was able to induce apoptosis through intrinsic and extrinsic pathways and was capable of decreasing sICAM-1, mTOR, cathepsin B concentrations, whereas increased Beclin-1 concentration was detected in both colon cancer cell lines. The novel compound represents promising multi-targeted potential in colorectal cancer, but further in vivo examinations are needed to confirm the claim.

## 1. Introduction

Colorectal cancer (CRC) represents a major health problem worldwide because of its mortality [1]. There is a list of predisposing risk factors that play an important part in CRC development. A positive family history and environmental lifestyle factors such as alcohol intake, smoking, bad dietary habits, including low intake of vegetables and fruits as well as high red meat intake, affect disease pathogenesis [2]. The risk for CRC might be also increased by specific bacterial species, such as *Bacteroides fragilis* [3].

Different classes of drugs are used to treat metastatic CRC and include adjuvant chemotherapy (5-fluorouracil or capecitabine, oxaliplatin, irinotecan), biologics (bevacizumab, aflibercept, and ramucirumab), salvage therapy drugs, immunotherapy, or targeted therapies [4,5]. The standard of care consists of two or three chemotherapeutic agents paired with biologics such as anti-VEGF agents or anti-EGFR drugs. The strategy of therapy in CRC depends on many patient-related and tumor-related factors [2]. The aim for the investigators is looking for the drugs that act at multiple targets. Such an approach represents a future perspective and allows to improve the efficacy of the therapy. The Food and Drug Administration approved encorafenib (BRAFTOVI, Array BioPharma Inc., Boulder, CO, USA) in combination with cetuximab for the treatment of adult patients with metastatic colorectal cancer (CRC) with a *BRAFV600E* mutation [6]. The key targets of encorafenib are the enzymes in the MAPK signaling pathway. The data demonstrated that it enhances TRAIL-induced apoptosis of colorectal cancer cells [7]. Considering the fact that sulfonamides are a classic group of antitumor agents with diverse pharmacological activity, researchers are designing novel chemical compounds containing this group. The sulfonamide moiety is a valuable element of the lead structures due to its pleiotropic action. These derivatives have the ability to inhibit the activity of phosphodiesterase type 5, carbonic anhydrase, tyrosinase, histone deacetylase, metalloproteinases, β-tubulin, or cyclin-dependent kinases [8,9,10,11], which may be important in the treatment of cancer. In many neoplastic diseases, including pancreatic, breast, colon, kidney, lung, and stomach cancer, the overexpression of two isoenzymes of carbonic anhydrase (CA IX and CA XII) are observed. Selective inhibition of isoforms IX and XII may lead to the initiation of programmed death of neoplastic cells, as well as reduce the risk of side effects of therapy, most often resulting from the undesirable inhibition of cytosolic forms [12,13]. The group of sulfonamides used in the treatment of cancer includes pazopanib, belinostat, dabrafenib, vemurafenib [14]. Pazopanib was approved in 2009 by the FDA for the treatment of advanced soft tissue sarcoma (STS) and kidney cancer. Antitumor activity is related to the inhibition of the activity of vascular endothelial growth factor receptor 1, -2, and -3 (VEGFR1-3), platelet endothelial growth factor receptor -α, and -β (PEGFRα, β), the stem cell factor receptor c-kit and fibroblast growth factor receptor (FGFR) [14]. Belinostat is a histone deacetylase inhibitor and was approved by the FDA in 2014 for the treatment of peripheral T-cell lymphoma [15,16]. In turn, dabrafenib and vemurafenib belong to BRAF inhibitors and are used in the treatment of melanoma with the *BRAFV600* mutation [17,18]. Among the sulfonamide derivatives described so far in the literature, the compound ABT-263 (Navitoclax, Selleckchem, Houston, TX, USA) deserves attention. This compound is at various stages of clinical trials in cancer treatment, which have confirmed its strong antitumor activity, as well as a significant improvement of standard chemotherapy activity. The mechanism of action is related to the inhibition of the activity of anti-apoptotic proteins from the Bcl-2 family [19,20,21]. A significant part of novel synthesized compounds are heterocyclic which contain at least one nitrogen in their ring structure. In this group triazine derivatives deserve attention. There can be distinguished three isomers: 1,2,3-triazine, 1,2,4-triazine, and 1,3,5-triazine basing on their nitrogen position in the ring system. Among 1,2,3-triazine only condensed v-triazines present promising anticancer properties. The biological activity of this compounds is associated with induction of apoptosis and inhibition of VEGFR-2, p70S6K, herpenase, JAK, Pim-1, and EGFR which play a crucial role in cancer resistance. In 2006 Anderson presented benzo[*d*][1,2,3]triazin-4-yl-(4-methoxy-phenyl)-methylamine, which exhibited marvelous anti-proliferative effect against four cancer cell lines: breast cancer (T-47D), colorectal cancer (HT-29), lung cancer (H1299), and melanoma (MDA-MB-435) [22]. Moreover, in the last ten years a huge number of 1,2,4-triazine derivatives have been synthesized. Currently novel compounds are at different stages of in vitro, in vivo, and clinical trials. The observed increased interest in this group of derivatives is related to their wide biological activity. Literature reports show that 1,2,4-triazines derivatives may inhibit: cyclin-dependent kinase, carbonic anhydrase IX and XII, SYK, EGFR, Wnt/β-catenin pathway, Hedgehog pathway, mTOR, RAS/RAF/MAPK. [23] The last group of triazine derivatives 1,3,5-triazine also play a crucial role in designing new anticancer drugs. In the literature s-triazine derivatives can be described as CDK, GSK-3β, Tie-2, SYK, carbonic anhydrase IX, VEGFR-2, mTOR, PI3K, histone deacetylase, dihydrofolate reductase, CK2 inhibitors (depending on the chemical structure) [24].

Recently, we proved that novel 7-methyl-5-phenyl-pyrazolo[4,3-*e*]tetrazolo[1,5-*b*][1,2,4]triazine sulfonamide derivatives possess promising anticancer properties [25]. In this research we present a novel 1,2,4-triazine derivative (**MM131**), which like encorafenib bears a pyrazole ring and belongs to the sulfonamide group with anticancer properties [23]. This prompted us to investigate the impact of these common features on the biological activity of the designed novel compound. Continuing our research, the aim of the study was to explain the anticancer effects of novel 1,2,4-triazine sulfonamide derivative (**MM131**) in DLD-1 and HT-29 colon cancer cells. The viability of cancer cells was checked by MTT assay and anti-proliferative effect was tested by DNA biosynthesis. The pro-apoptotic potential of the novel derivative was confirmed by flow cytometry and fluorescence microscopy. Induction of apoptosis was analyzed using two independent methods like Annexin V binding assay and ethidium bromide/acridine orange staining. The activity of caspases, which are engaged in the extrinsic (caspase-8) and intrinsic (caspase-9) apoptotic pathway was determined. The activity of caspase-3/7 was also demonstrated. Cell cycle analysis was checked by flow cytometry. Additionally, the concentration of important proteins (sICAM-1, mTOR, Beclin-1, cathepsin B) involved in the pathogenesis and poor prognosis of colorectal cancer was also evaluated by ELISA.

## 2. Results

### 2.1. Chemistry

**Scheme 1:**
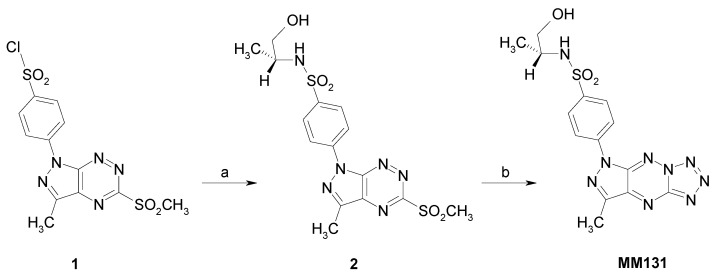
Reagents and conditions: (**a**) MeCN, rt; (**b**) NaN_3_, EtOH, reflux.

The starting material for the preparation of the new tricyclic sulfonamide **MM131** was the corresponding chlorosulfone derivative **1** (Scheme 1) which was obtained by multistage synthesis according to the procedures described in the literature [25,26,27,28,29,30,31]. Thus, obtained compound **1** was mixed with the (*R*)-enantiomer of 2-amino-propan-1-ol in anhydrous acetonitrile at room temperature. The progress of the conversion was monitored by TLC. After complete consumption of the substrate **1**, product **2** was isolated and purified by column chromatography and then subjected to spectroscopic analysis in order to confirm its chemical structure. In the last step compound **2** was subjected to nucleophilic substitution reaction with sodium azide to replace the methylsulfanyl group. As a result of this transformation, in the first step the corresponding intermediate 5-azido-pyrazolo-[4,3-*e*][1,2,4]triazine was formed which under the reaction conditions undergoes intramolecular cyclization to give the final tricyclic sulfonamide **MM131**. The structure and the purity of the newly synthesized compound **MM131** were characterized using spectroscopic methods (^1^H- and ^13^C-NMR, and HRMS) together with elemental analysis.

### 2.2. Biological Studies

**Figure 1:**
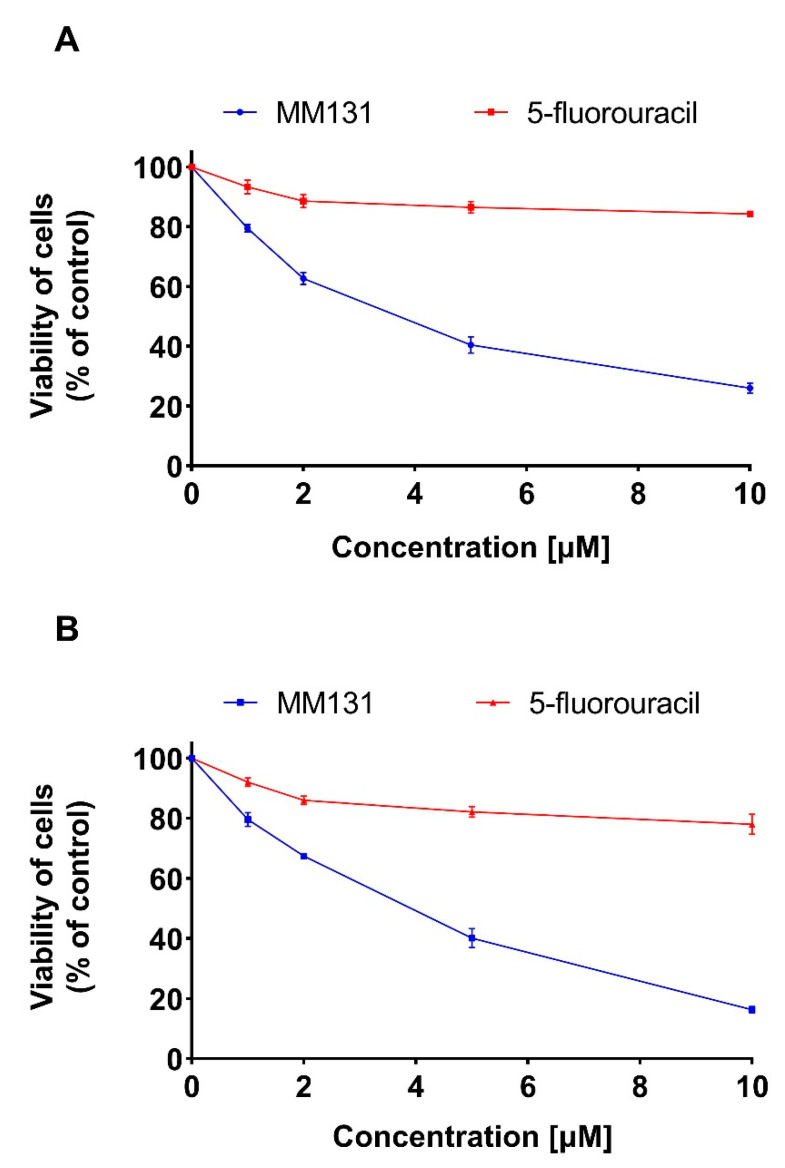

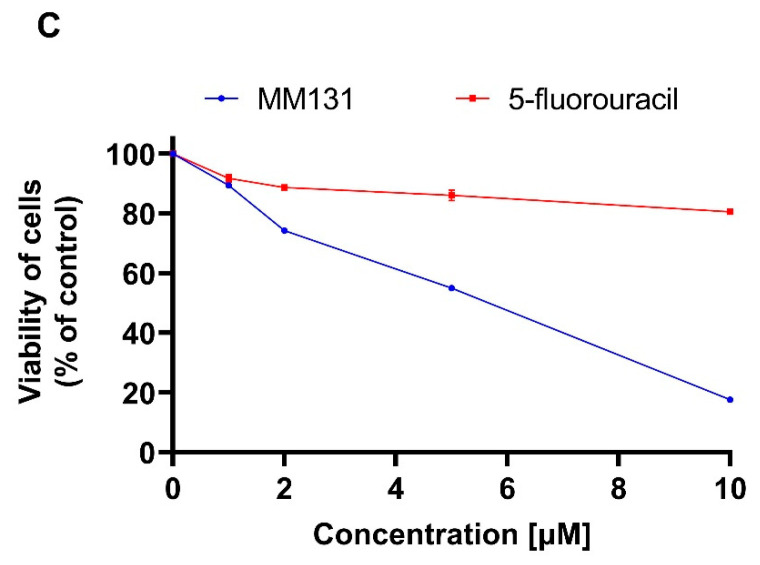
Cell viability was detected by MTT assay. The graph shows the cytotoxicity profile of **MM131** and 5-fluorouracil against DLD-1 cells, (**A**) HT-29 cells, (**B**) and human skin fibroblasts; (**C**) at different concentrations (1–10 μM) at 24 h of incubation. The viability of colorectal cancer cells is represented as a percentage of untreated cells (± SEM, *n* = 3).

Cytotoxicity of the tested compounds (**MM131**, 5-fluorouracil) towards two human colon cancer cell lines and human skin fibroblasts was evaluated using varying concentrations for 24 h. **MM131** exhibited significant dose-dependent cytotoxicity on both cell lines with very similar efficacy. After 24 h exposure, 50% of DLD-1 cells died at 3.4 µM (Figure 1A) and 50% of HT-29 cells at 3.9 µM (Figure 1B). IC_50_ for 5-fluorouracil was 151 µM in DLD-1 and 150 µM in HT-29 cells (data not shown). **MM131** was not so effective in decreasing the viability of human skin fibroblasts compared to both colon cancer cells. Its IC_50_ was reached at 5.65 µM (Figure 1C).

**Figure 2:**
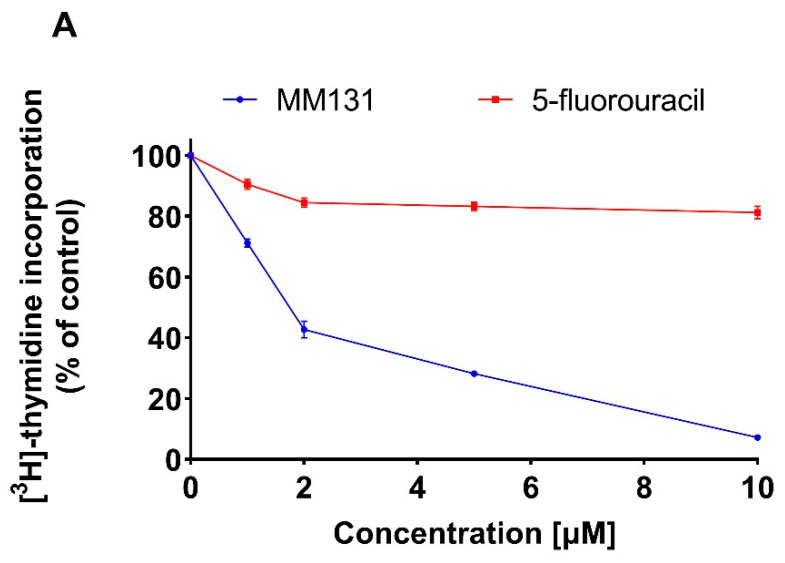

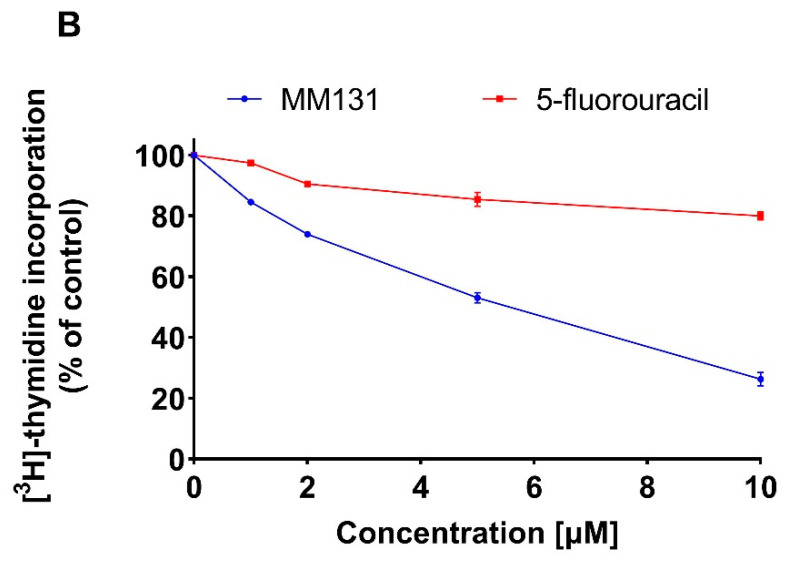
[^3^H]-thymidine incorporation assay was used to assess the anti-proliferative effect of **MM131** and 5-fluorouracil against DLD-1 (**A**) and HT-29 (**B**) cells at different concentrations (1–10 μM) at 24 h of incubation. The results are presented as a percentage of untreated cells (± SEM, *n* = 3).

The anti-proliferative potential was analyzed by the incorporation of radioactive [^3^H]-thymidine into the DNA of colon cancer cells after 24 h incubation with various concentrations of the tested agents. The results from the experiment are shown in Figure 2. We proved that the IC_50_ value for **MM131** was 1.7 µM in DLD-1 colon cancer cells and 5.6 µM in HT-29 colon cancer cells. IC_50_ for fluorouracil was higher than 10 µM in both tested colon cancer cells.

**Figure 3:**
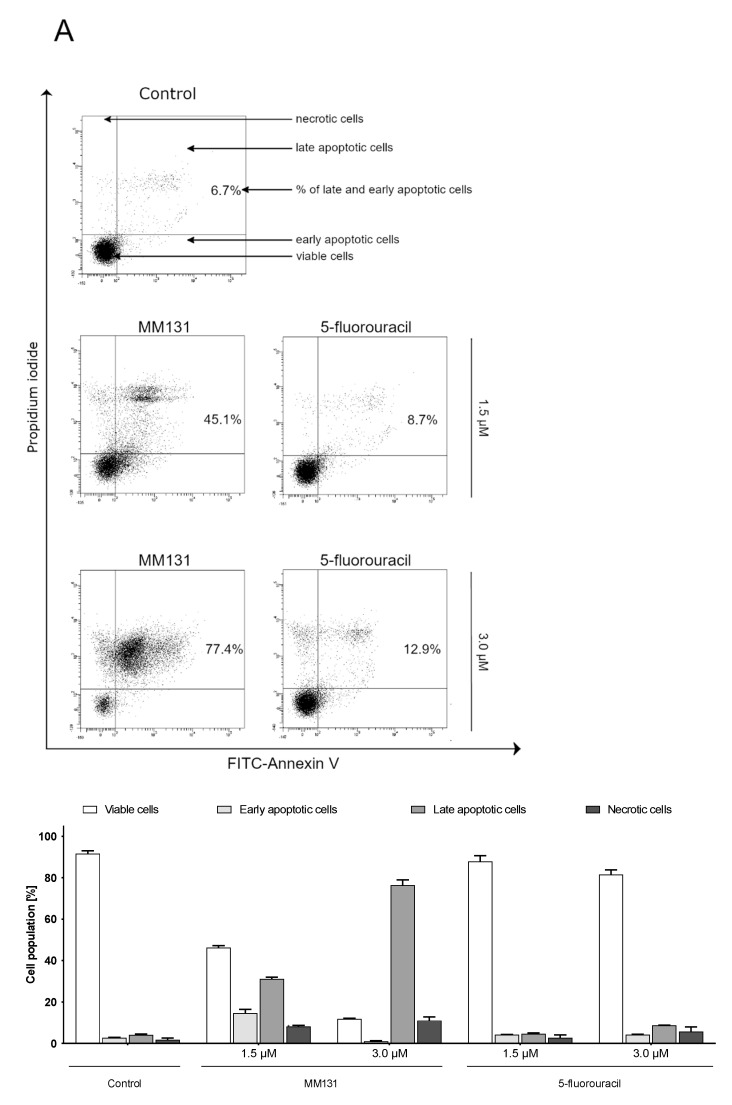

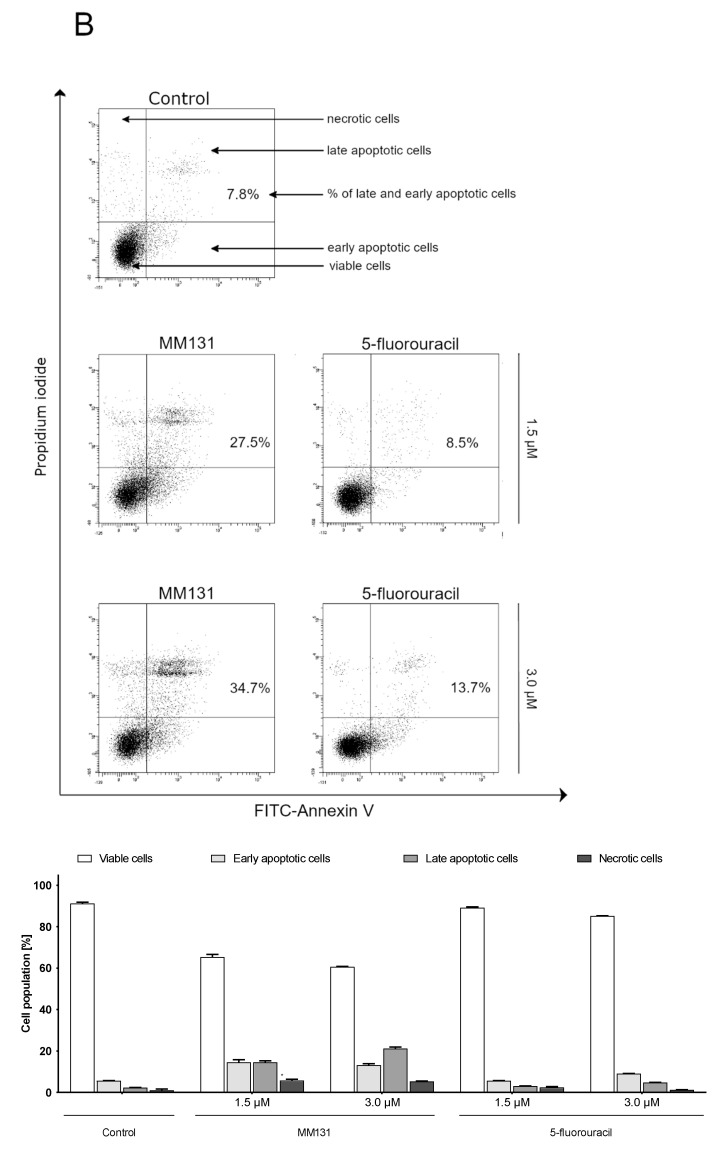
Apoptosis measurement by flow cytometry with Annexin V-FITC staining in DLD-1 (**A**) and HT-29 (**B**) exposed for 24 h to **MM131** and 5-fluorouracil. Left bottom square has live cells, right bottom early apoptotic cells, the superior left square contains necrotic cells, and superior right late apoptotic cells. The apoptotic rate of the compounds is presented as a sum of mean percentage of early- and late-apoptotic cells in the adequate cell line (*n* = 3 in duplicates).

**Figure 4:**
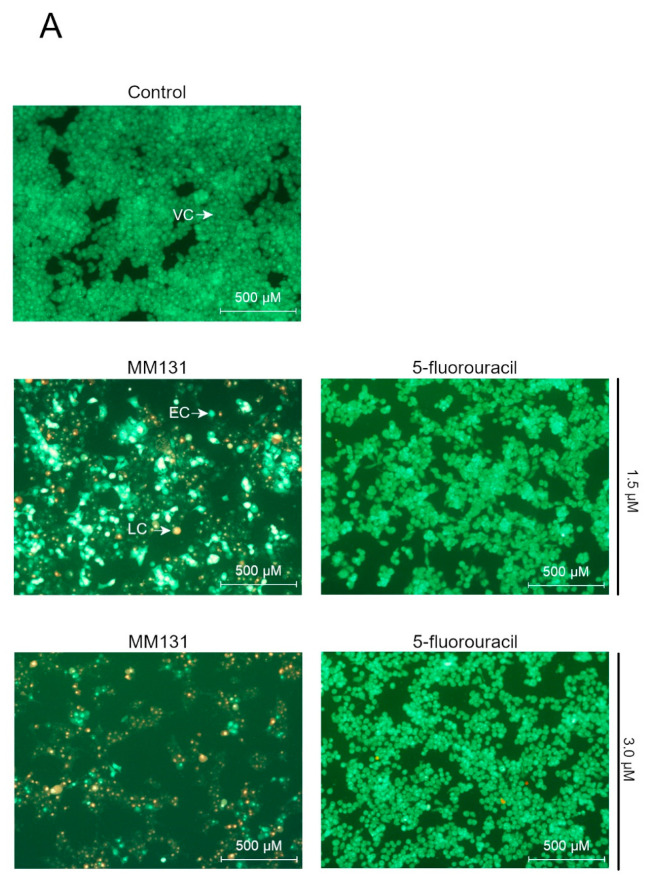

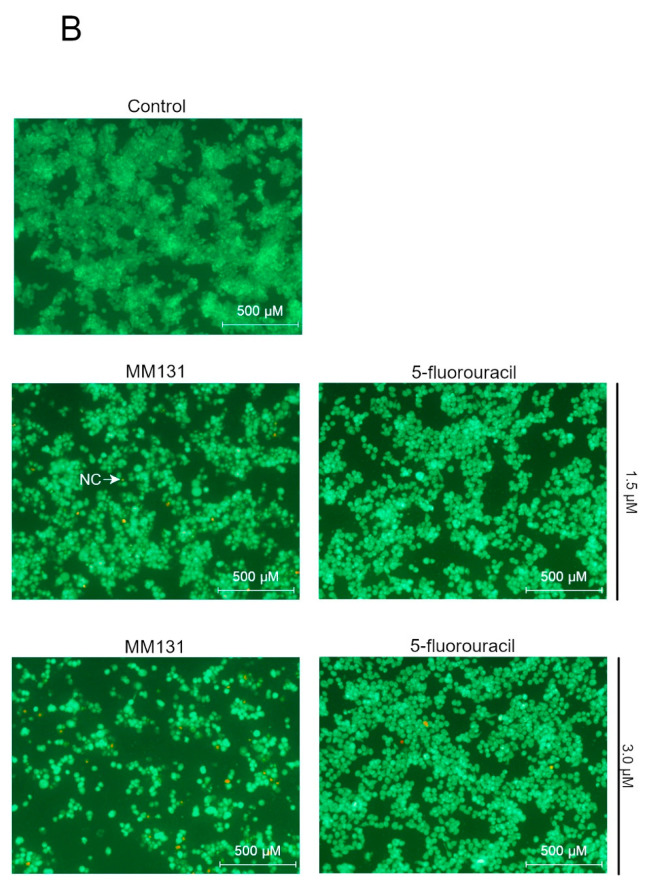
Visualization of morphological specificity of colorectal cancer cells DLD-1 (**A**) and HT-29 (**B**) exposed to **MM131** and 5-fluorouracil for 24 h of incubation stained with acridine orange and ethidium bromide. Arrows and abbreviations indicate formation of: VC—viable cells, EC—early apoptotic cells, LC—late apoptotic cells, NC—necrotic cells. Magnification 100×.

The induction of apoptosis after treatment with **MM131** and 5-fluorouracil was tested by two independent methods such as Annexin V binding assay as well as ethidium bromide/acridine orange staining. The results are presented in Figure 3 and Figure 4.

Analysis revealed that **MM131** was five times stronger in inducing apoptosis than 5-fluorouracil at a dose of 1.5 µM and six times stronger compared to the reference compound at a dose of 3 µM in DLD-1 colon cancer cells. As can be seen from Figure 3A, the percentages of early- and late apoptotic cells were 45.1 % and 77.4% for **MM131** at doses of 1.5 and 3 µM, whereas 8.7% and 12.9% of early- and late apoptotic cells were detected after treatment with 5-fluorouracil.

The effect of the compounds on apoptosis activation in HT-29 colon cancer cells was demonstrated in Figure 3B. It was proved again that **MM131** was more effective inducer of programmed cell death than 5-fluorouracil. The results showed that **MM131** led to 27.5% (1.5 µM) and 34.7% (3 µM) of early- and late apoptotic cells in the analyzed population of HT-29 cells. Analysis of the pro-apoptotic effect of 5-fluorouracil revealed 8.5% and 13.7% of apoptotic cells.

Observations from the flow cytometry were confirmed by a second method and are shown in Figure 4. Specific staining with ethidium bromide and acridine orange allowed us to distinguish viable, dead, and apoptotic cells. Viable cells were indicated as dark green fluorescence. Bright green and orange fluorescence represented early- and late-apoptotic cells. Necrotic cells were visible as red fluorescence. A significant number of early- and late apoptotic cells were observed after 24 h of incubation with **MM131**, where bright green and orange are dominant, especially in DLD-1 colon cancer cells.

**Figure 5:**
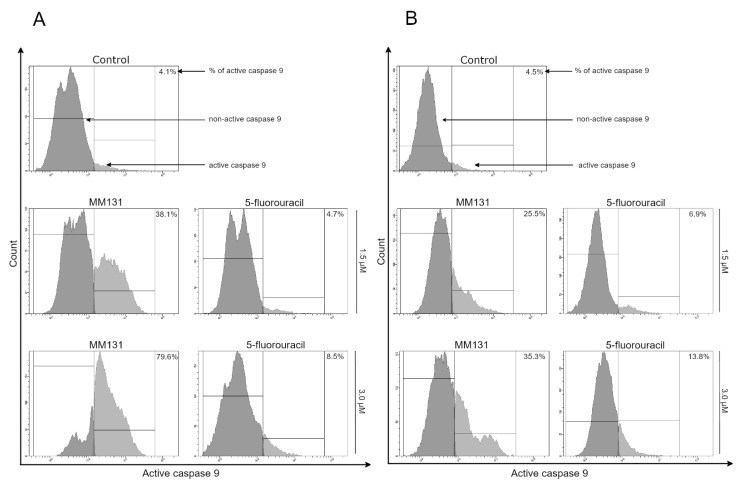
Flow cytometric analysis of caspase-9 activity in DLD-1 (**A**) and HT-29 (**B**) colorectal cancer cells after 24 h incubation with **MM131** and 5-fluorouracil. The number of cells with active caspase-9 is represented as a mean of three separate experiments.

Caspase-9 activity was checked to confirm that **MM131** activated programmed cell death via the mitochondrial pathway (Figure 5). In the control sample, we detected only 4.1% of cells with active caspase-9. After exposition of DLD-1 cells to **MM131** at 1.5 µM and 3 µM concentrations, we demonstrated 38.1% and 79.6% of DLD-1 cells with active caspase-9, while 5-fluorouracil led to the 4.7% and 8.5% of cells with active caspase-9.

The similar situation was demonstrated in the HT-29 cell line, but the effect was weaker than in DLD-1 colon cancer cells. In the analyzed population of HT-29 cells we observed 6.9% cells with active caspase-9 after treatment with 5-fluorouracil (1.5 μM), whereas exposition to **MM131** caused that 25.5% of cells had active caspase-9. Incubation with a higher dose of compounds activated caspase-9 more efficiently. We demonstrated that 35.3% and 13.8% of cells had active caspase-9 after incubation with **MM131** and 5-fluorouracil.

**Figure 6:**
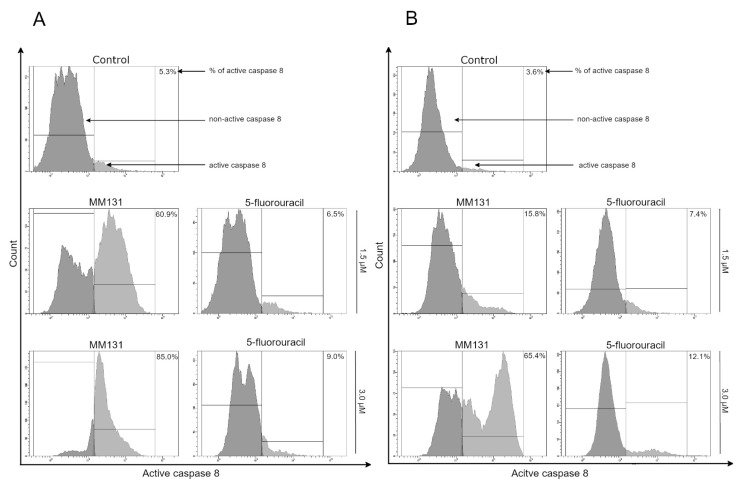
Flow cytometric analysis of caspase-8 activity in DLD-1 (**A**) and HT-29 (**B**) colorectal cancer after 24 h of incubation with **MM131** and 5-fluorouracil. The number of cells with active caspase-8 is represented as a mean of three separate experiments.

The extrinsic apoptotic pathway can be confirmed by the activity of caspase-8. We checked it after 24 h of incubation of DLD-1 and HT-29 colon cancer cells with 1.5 and 3 µM of **MM131** and a reference compound (Figure 6). In analyzed control sample, we detected 5.3% of DLD-1 cells that possessed active caspase-8. **MM131** led to the activation of caspase-8 in colon cancer cell lines. We showed 60.9% and 85% of DLD-1 cells with active caspase-8, which were exposed to 1.5 µM and 3 µM concentrations of **MM131**. The effect of 5-fluorouracil was nine times weaker in comparison with **MM131** in both tested doses in DLD-1 colon cancer cells.

However, treatment with **MM131** resulted in the activation of caspase-8 in HT-29 colon cancer cells, but the effect was not so significant like in DLD-1 colon cancer cells. Analysis from flow cytometry revealed that there were 15.8% and 65.4% of HT-29 cells with active caspase-8 after 24 h incubation with **MM131** at doses of 1.5 and 3 µM. After incubation with 5-fluorouracil (1.5 and 3 µM) we observed only 7.4% and 12.1% of HT-29 cells with active caspase-8.

**Figure 7:**
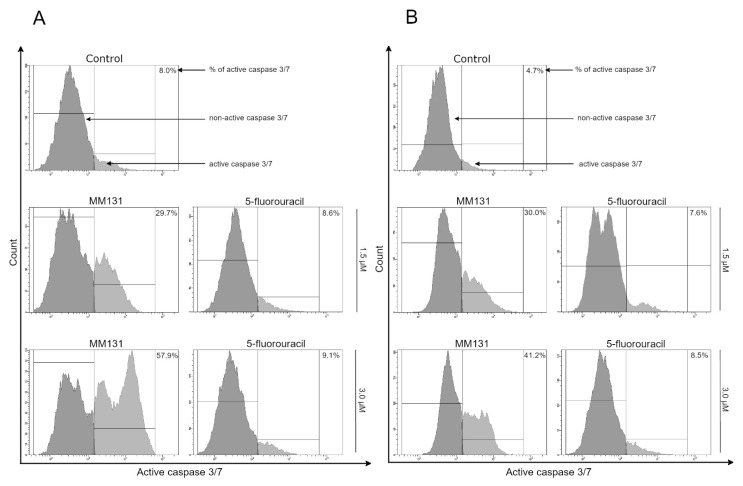
Caspase-3/7 activity utilizing flow cytometry analysis following 24 h of incubation with **MM131** and 5-fluorouracil in DLD-1 (**A**) and HT-29 (**B**) colorectal cancer cells. The total number of cells with active caspase-3/7 is demonstrated as a mean of three separate experiments.

Finally, the activity of caspase-3/7 was analyzed in both tested colon cancer cell lines after treatment with 5-fluorouracil and the novel **MM131** (Figure 7). The highest percentage of colon cancer cells with active caspase-3/7 was determined after exposition to **MM131** at a dose of 3 µM. We established 57.9% of DLD-1 cells and 41.2% of HT-29 cells with active caspase-3/7. The activation of effector caspase was stronger than 5-fluorouracil.

**Figure 8:**
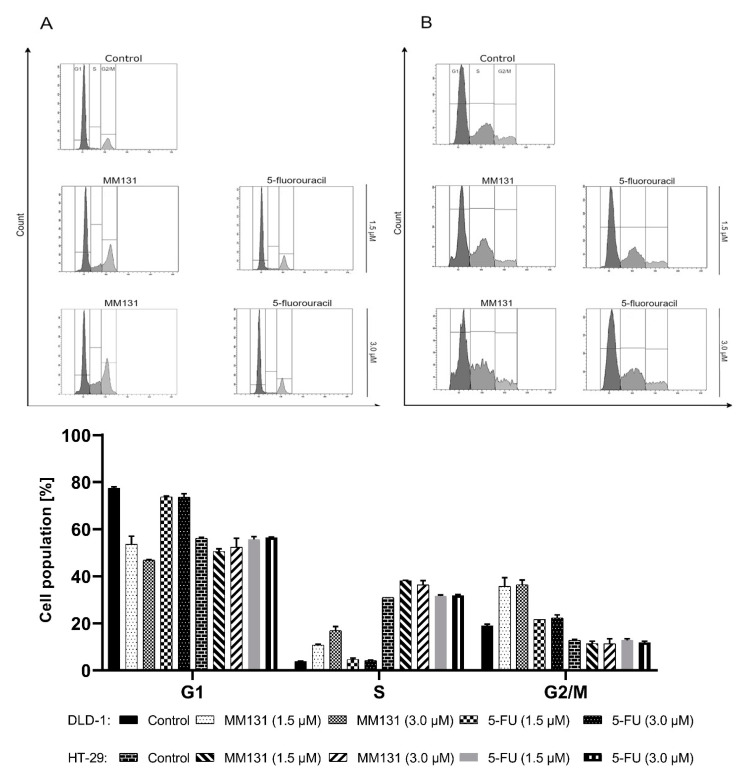
The influence of the tested compounds on cell cycle distribution in human colon DLD-1 (**A**) and HT-29 cells (**B**) after 24 h of incubation. Representative flow cytometry profiles depicting the cell cycle distribution and histograms representing the percentages of the cell population at each phase of the cell cycle are demonstrated. Mean ± SD from three independent experiments (*n* = 3) done in duplicate are presented.

Cell cycle analysis revealed that **MM131** led to the accumulation of DLD-1 cells in the S phase and G_2_/M of the cell cycle (Figure 8). The percentage of cells in the S phase increased from 3.75% in the untreated DLD-1 control cells to 10.7% and 16.85% after treatment with **MM131** (1.5 μM and 3 μM, respectively). Additionally, the percentage of DLD-1 cells in the G_2_/M phase also increased from 18.9% in the control cells to 35.65% and 36.4% after incubation with **MM131** (1.5 μM and 3 μM, respectively). After exposition of HT-29 cells to the novel compound, we demonstrated that **MM131** was specific to only one S phase of the cell cycle. The percentage of cells in the S phase increased from 31% in the untreated HT-29 control cells to 38.1% and 36.4% after treatment with **MM131** (1.5 and 3 μM, respectively).

**Figure 9:**
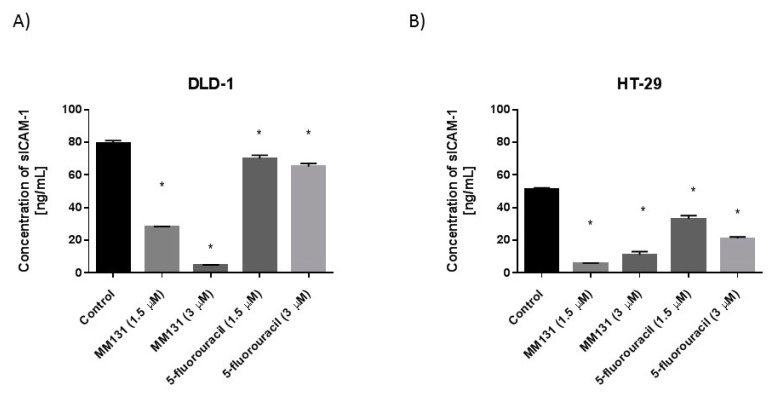
Quantification of sICAM-1 by ELISA. Bar graphs demonstrating sICAM-1 concentration in media from DLD-1 (**A**) and HT-29 (**B**) colorectal cancer cells after 24 h of incubation with **MM131** and 5-fluorouracil. *n* = 3 in duplicates; * *p* < 0.05 versus respective control group.

In the next stage of the experiment we checked the concentration of sICAM-1 in the samples after 24 h incubation (Figure 9). We detected 51 ng/mL of sICAM-1 in the control sample of HT-29, whereas exposition to the tested compounds led to decrease in the analyzed protein concentration. After 24 h of incubation with **MM131** at 1.5 µM, we observed 5.5 ng/mL of sICAM-1. A higher dose of **MM131** (3 µM) resulted in 11 ng/mL of sICAM-1. The reference compound was not so efficient in decreasing the analyzed protein. We observed 33 ng/mL of sICAM-1 after 24 h of incubation with 5-fluorouracil (1.5 µM) and 21 ng/mL of sICAM-1 after 24 h exposure to 5-fluorouracil (3 µM).

In DLD-1 colon cancer cells we found higher levels of sICAM-1 in the control sample in comparison with untreated HT-29 cells. The concentration of sICAM-1 was 79 ng/mL. 5-fluorouracil (1.5 and 3 µM) reduced the concentration of sICAM-1 to 70 and 65 ng/mL. The values were statistically significant. The most significant decrease was shown after 24 h of incubation with the novel derivative (**MM131**). The lower dose of the tested compound decreased the level of sICAM-1 to 28.2 ng/mL while the higher dose of **MM131** decreased the concentration of sICAM-1 to 4.7 ng/mL.

**Figure 10:**
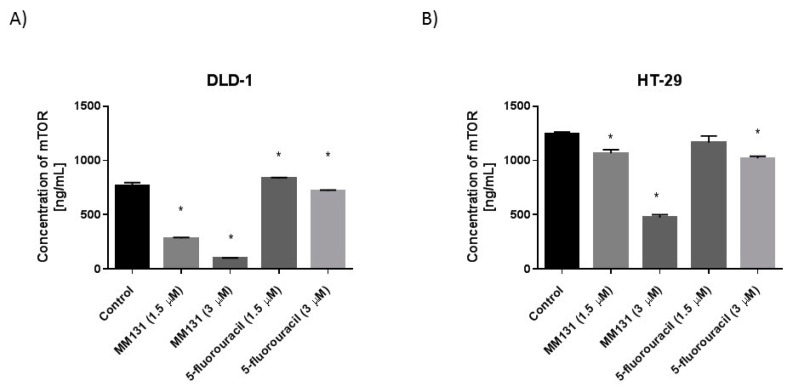
Quantification of mTOR by ELISA. Bar graphs demonstrating mTOR concentration in DLD-1 (**A**) and HT-29 (**B**) colorectal cancer cells after 24 h of incubation with **MM131** and 5-fluorouracil. *n* = 3 in duplicates; * *p* < 0.05 versus respective control group.

In the next stage of the research, mTOR concentration was also analyzed after 24 h of incubation with 1,2,4-triazine novel derivative (**MM131**) and the reference compound (Figure 10). We demonstrated the most significant decrease of mTOR concentration after 24 h of incubation of HT-29 cells with **MM131** at a 3 µM concentration. The value was 475 ng/mL compared to the control, where we measured 1241 ng/mL. In case of exposition to fluorouracil (1.5 and 3 µM), we detected 1163 ng/mL and 1019 ng/mL, respectively.

In DLD-1 cells we observed a similar effect when we analyzed **MM131**. It was the most efficient in decreasing of mTOR concentration in cell lysates in comparison with the control sample. **MM131** at a dose of 3 µM decreased the concentration from 763 ng/mL in untreated cells to 99 ng/mL in DLD-1 cells. 5-fluorouracil at a dose of 1.5 µM increased the concentration of mTOR, whereas at a higher dose decreased the concentration to 718 ng/mL. All values were statistically significant.

**Figure 11:**
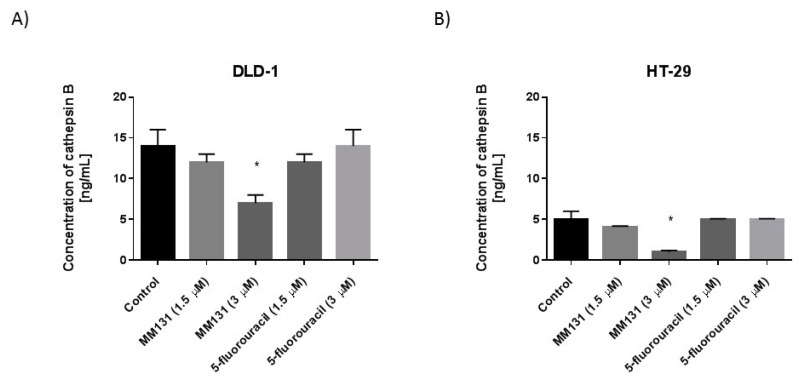
Quantification of cathepsin B by ELISA. Bar graphs demonstrating cathepsin B concentration in DLD-1 (**A**) and HT-29 (**B**) colorectal cancer cells after 24 h of incubation with **MM131** and 5-fluorouracil. *n* = 3 in duplicates, * *p* < 0.05 versus respective control group.

We checked cathepsin B levels in HT-29 and DLD-1 cells (Figure 11). In both cases, we observed a significant decrease in cathepsin B concentration after 24 h of incubation with **MM131** at a dose of 3 µM. In HT-29 cells we detected 1 ng/mL and in DLD-1 we proved that the concentration of cathepsin B was 7 ng/mL. Both values were statistically significant in comparison with the control sample. 5-fluorouracil had no significant effect cathepsin B concentration in both tested cancer cell lines.

**Figure 12:**
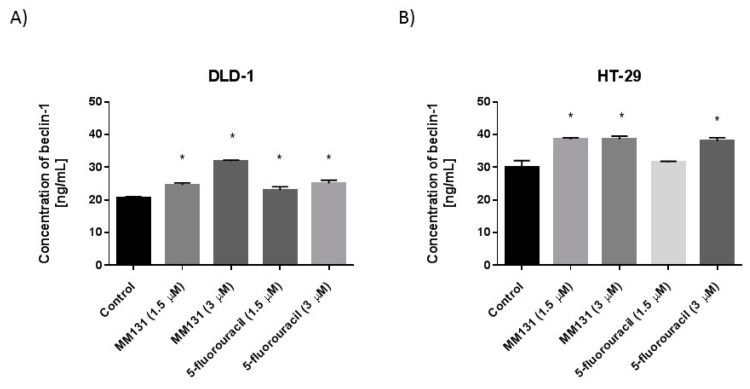
Quantification of Beclin-1 by ELISA. Bar graphs demonstrating Beclin-1 concentration in DLD-1 (**A**) and HT-29 (**B**) colorectal cancer cells after 24 h of incubation with **MM131** and 5-fluorouracil. *n* = 3 in duplicates; * *p* < 0.05 versus respective control group.

The concentration of Beclin-1 was also analyzed, and the results are presented in Figure 12. It was proved that 5-fluorouracil (3 µM) as well as **MM131** (1.5 and 3 µM) caused an increase in Beclin-1 concentration in HT-29 colon cancer cells. We showed that the concentration of Beclin-1 was 38.5 ng/mL after treatment with **MM131** at both tested doses. In DLD-1 cells, we established that **MM131** increased the level of Beclin-1 from 20.5 ng/mL in untreated cells to 24.6 ng/mL after exposition to the lower dose of **MM131** and 31.8 ng/mL after 24 h of incubation with the higher dose of the novel derivative. Similarly, 5-fluorouracil also increased the level of Beclin-1, but it was not as strong as **MM131**.

## 3. Discussion

The discovery of defects in cell death signaling has become a key reason in the search for new therapeutic approaches. Targeting cell death in colorectal cancer represents a major direction in therapy [32]. A number of clinical investigations are due to target proteins involved in extrinsic and intrinsic apoptotic pathways such as APAF1, cytochrome c, tBID, FADD, TNF, TRAIL, and IAPs. The anti-apoptotic Bcl-2 proteins represent important targets for various small molecules, which release a multidomain of Bcl-2 proteins and induce cell death. The promising results from in vitro studies were obtained after use of ABT-263. Its mechanism of action promotes cell death and inhibits ERK kinase 1/2 [33]. Oblimersen as a representant of antisense technology was also tested in patients with metastatic colorectal cancer. Its combination with irinotecan in a phase I trial has shown safety, but finally it was not approved for treatment. IAPs (XIAP and survivin) are overexpressed in colorectal cancer and inhibit caspases activity. XIAP was a target for antisense oligonucleotide (AEG35156). The efficacy of embelin was also tested, whose targets were surviving, Bcl-2 and Bcl-xL. Additionally, survivin peptide vaccine was considered to be a very potent immunotherapeutic regimen and the results from randomized phase II trial in patients with HLA A24-positive pancreatic adenocarcinoma were recently published [34]. The literature data presented above confirm the hypothesis that the induction of apoptosis is an attractive molecular target in anti-neoplastic therapy. Our research is in line with the trend of looking for compounds that induce programmed cell death.

Our study has demonstrated that DLD-1 and HT-29 colorectal cancer cell cultures treated with different concentrations of **MM131** (1.5 and 3 µM) undergo apoptosis. The mechanism refers to the activity of caspase-8, caspase-9, and caspase-3/7 thus confirming that extrinsic and intrinsic apoptotic pathways were induced during programmed cell death. We proved that **MM131** led to the accumulation of DLD-1 cells in the S phase and G_2_/M of cell cycle. After exposition of HT-29 cells to novel compound, we demonstrated that **MM131** was specific to only one S phase of the cell cycle.

Mechanistic target of rapamycin (mTOR) activation is responsible for the promotion of tumor growth and metastasis. Some mTOR inhibitors have been approved by the FDA to treat cancer, but a high number of them are still under investigation in many clinical trials [35,36,37]. Our analysis revealed that the novel **MM131** was able to reduce mTOR concentrations in both colon cancer cell lines after 24 h incubation in comparison with untreated cells and the inhibitory effect was much stronger than the reference compound.

Another important protein is soluble intercellular adhesion molecule-1 (sICAM-1), which is elevated in the sera of different cancers and is considered to be a prognostic marker in patients with colorectal cancer. Schellerer et al. proved that elevated sICAM-1 concentrations are linked to significantly decreased overall and cancer-related survival [38]. We noticed significantly reduced levels of sICAM-1 after 24 h of incubation with **MM131** in DLD-1 as well as HT-29 cells.

Our attention was also focused on Beclin-1. Its downregulation is connected to cancer progression and metastasis [39]. Zhang et al. examined the effect of Beclin-1 overexpression on aggressive phenotypes of colon cancer cells. They suggested that Beclin-1 overexpression may improve the differentiation of HCT 15 and HCT 116 cells and suppressed colon cancer tumor growth by increasing autophagy and apoptosis [40]. The study was evidence that Beclin-1 acts as a molecule target for the treatment of colon cancer. In our experiment, we determined Beclin-1 levels and showed that **MM131** efficiently increased Beclin-1 concentrations in both DLD-1 and HT-29 colon cancer cells.

Cathepsin B is an endopeptidase, whose increased levels were documented in many cancers, for example, in gliomas, melanomas, prostate cancer, and breast cancer [41,42,43,44,45]. Campo et al. demonstrated a positive correlation between elevated cathepsin B levels and the shortened survival of patients with CRC [46]. Many studies in the literature have shown a link between higher cathepsin B levels with enhanced invasion and angiogenesis [47]. There is an urgency to develop clinically useful agents that will be able to inhibit the action of cathepsin B. Our research demonstrated that **MM131** at a dose of 3 µM significantly reduced cathepsin B concentrations in DLD-1 and HT-29 colon cancer cells.

The ability of compounds to inhibit more than one specific molecular target represents a promising strategy in anticancer treatment. Recently, we examined the multi-targeted potential of a monoclonal antibody against mucin 1 (MUC1) and a novel octahydropyrazino[2,1-a:5,4-a′]diisoquinoline derivative (OM-86II) in estrogen receptor-positive MCF-7 human breast cancer cells [48]. It was demonstrated that anti MUC1 antibody with OM-86II decreased MMP-2, MMP-9, sICAM-1, mTOR, TNF-α, and COX-2 concentrations. The combination of compounds exerted the pro-apoptotic potential and induced G_2_/M cell cycle arrest in MCF-7 cells. In this study, we proved that the novel **MM131** with its pro-apoptotic activity and capability to inhibit more than one specific molecule represents a promising therapeutic approach in colorectal cancer, but further in vivo examinations are needed to confirm the claim.

## 4. Materials and Methods

### 4.1. **MM131** Synthesis

#### 4.1.1. General

Melting points were determined on a Mel-Temp apparatus and were uncorrected. ^1^H- and ^13^C-NMR spectra were recorded on a Varian spectrometer (400 MHz for ^1^H- and 100 MHz for ^13^C-, Siedlce, Poland). The chemical shift values were expressed in ppm (part per million) with tetramethylsilane (TMS) as an internal reference. The relative integrals of peak areas agreed with those expected for the assigned structures. The molecular weight of the final compounds was assessed by electrospray ionization mass spectrometry (ESI/MS) on Thermo Scientific^TM^ Q Exactive^TM^ Mass Spectrometer Series (Warsaw, Poland). Elemental compositions were within ± 0.4% of the calculated values. For the preparation and spectroscopic data of compound **1**, see the literature [25].

#### 4.1.2. Synthesis of *N*-(*R*)-(1-hydroxypropan-2-yl)-4-(3-methyl-5-methylsulfonyl-1*H*-pyrazolo[4,3-*e*][1,2,4]triazyn-1-yl)benzenesulfonamide (**2**)

A mixture of chlorosulfonyl chloride (**1**) (194 mg, 0.5 mmol) and (*R*)-2-aminopropan-1-ol (112 mg, 1.5 mmol) in anhydrous acetonitrile (5 mL) was stirred overnight at room temperature, and then the reaction mixture was concentrated in vacuo to afford the crude sulfonamide, as a yellow solid. The residue was purified on silica gel using a mixture of CH_2_Cl_2_:EtOH (25:1) as eluent to give the titled compounds as a yellow solid.

Yield 75%. Melting point: 215–219 °C; ^1^H-NMR (CD_3_OD) δ: 1.03 (d, 3H, *J* = 6.4 Hz), 2.85 (s, 3H), 3.33–3.36 (m, 2H), 3.46 (q, 1H, *J* = 5.8 Hz) 3.58 (s, 3H), 4.57 (bs, 1H, OH), 8.13 (d, 2H, *J* = 8.8 Hz), 8.65 (d, 2H, *J* = 8.8 Hz); ^13^C-NMR (CD_3_OD) δ: 11.13, 17.98, 41.05, 52.55, 66.90, 121.35, 129.62, 139.31, 141.44, 142.31, 147.73, 149.90, 162.88; HRMS (ESI, *m*/*z*) Calcd for C_15_H_18_N_6_O_5_S_2_ [M^+^ + H] 427.08529. Found [M^+^ + H] 427.08593. Anal. Calcd for C_15_H_18_N_6_O_5_S_2_: C, 42.24; H, 4.25; N, 19.71. Found: C, 42.10; 4.37; N, 19.59. (Compound **2** from Supplementary Materials)

#### 4.1.3. Synthesis of Tricyclic Sulfonamides (**MM131**)

Sulfonamide derivative **2** (140 mg, 0.33 mmol) was dissolved in anhydrous ethanol (5 mL), and sodium azide (21 mg, 0.33 mmol) was added. The reaction mixture was refluxed until the substrate disappeared (control TLC). Then, the solvent was evaporated and the crude product was purified using column chromatography and CH_2_Cl_2_:MeOH (50:1) mixture as eluent to give the final compounds as a yellow solid.

*N*-(*R*)-(1-hydroxypropan-2-yl)-4-[7-methyl-5*H*-pyrazolo[4,3-*e*]tetrazolo[1,5-*b*][1,2,4]-triazin-5-yl)]benzenesulfonamide (**MM131**): Yield 91%. Melting point: 197–202 °C; ^1^H-NMR (CD_3_OD) δ: 1.01 (d, 3H, *J* = 6.4 Hz), 2.85 (s, 3H), 3.27–3.35 (m, 2H), 3.45 (q, 1H, *J* = 5.6 Hz), 4.57 (s, 1H, OH), 8.10 (d, 2H, *J* = 8.8 Hz), 8.45 (d, 2H, *J* = 8.8 Hz); ^13^C-NMR (CD_3_OD) δ: 12.03, 18.82, 53.37, 67.74, 121.08, 130.53, 141.54, 142.80, 144.19, 149.15, 149.70, 150.18; HRMS (ESI, *m*/*z*) Calcd for C_14_H_15_N_9_O_3_S [M^+^ + H] 390.10913. Found [M^+^ + H] 390.10926. Anal. Calcd for C_14_H_15_N_9_O_3_S: C, 43.18; H, 3.88; N, 32.37. Found: C, 43.00; H, 4.06; N, 32.25. (Compound **MM131** from Supplementary Materials)

### 4.2. Cell Culture of HT-29 and DLD-1 Cells

Cell culture HT-29 (HTB-38), DLD-1 (CCL-221) human colorectal adenocarcinoma cell lines were acquired from the ATCC—American Type Culture Collection (ATCC, Manassas, VA, USA). The first cell line was grown in McCoy’s 5a medium (Pan Biotech., Aidenbach, Lower Bavaria, Germany), the second cell line was maintained in RPMI 1640 medium (ATCC, Manassas, VA, USA). The growth supplement for the cell culture (Fetal Bovine Serum—FBS (Eurx, Gdansk, Poland) and the antimicrobial substances (penicillin/streptomycin—Corning, Kennebunk, ME, USA) were added in 10% and 1% concentration, respectively. The incubator asserted the appropriate growth conditions that are required for both cell lines: 5% of carbon dioxide, 37 °C and humidity between 90–95%. To culture the cells 100 mm plates were used. After a cell line reached about 80–90% confluency, the detachment of cells with 0.05% trypsin containing 0.02% EDTA (Corning, Kennebunk, ME, USA) and PBS (Corning, Kennebunk, ME, USA) was performed. The cells were then reseeded at a density 5 × 10^5^ of cells per well in a six-well plate in 1 mL of the appropriate medium and after 24 h incubation used in the presented tests [25].

### 4.3. Cell Viability Assay

The cytotoxic effect of **MM131** on both colorectal cell lines was measured by MTT assay. 5-flurouracil (Sigma-Aldrich, St Louis, MO, USA) was used as a reference drug. The cells were incubated with a serial dilution of the tested compounds and the reference drug for 24 h in duplicates. Then the liquid above the cells was removed by aspiration and the cells were rinsed three times with Phosphate-Buffered Saline (Corning, Kennebunk, ME, USA) without calcium and magnesium at room temperature. Afterwards, 50 µL of 5 mg per mL of MTT was added to 1 mL of PBS. After the required time elapsed, the MTT solution was removed, and the formazan crystals were dissolved in 1 mL of DMSO (Sigma-Aldrich, St Louis, MO, USA). A wavelength of 570 nm was used to measure the absorbance with Spectrophotometer UV-VIS Helios Gamma (Unicam/ThermoFisher Scientific Inc., Cleveland, OH, USA). The obtained absorbance in the control cells (without compound) was taken as 100%, while the survival of the cells incubated with the tested compounds was presents as a percentage of the control value [49].

### 4.4. [^3^H]-Thymidine Incorporation Assay

The anti-proliferative effect of the novel synthesized compound was performed by [^3^H]-thymidine incorporation assay as described in the literature [50]. The cells were exposed to various concentrations of the novel 1,2,4-triazine sulfonamide derivative and the reference drug for 24 h. Following the incubation, the cells were washed with Phosphate-Buffered Saline (PBS - Corning, Kennebunk, ME, USA) without calcium and magnesium and fresh medium was added. Then 0.5 µCi of radioactive [^3^H]-thymidine was appended, and the incubation was continued for four hours. In the first step the liquid was expelled, and the cells were placed on ice and washed two times with 1mL of 0.05 M Tris-HCl buffer comprising of 0.11 M NaCl and two times with 1 mL of 5% TCA (Stanlab, Lublin, Polska) acid. Finally, the cells were solubilized at room temperature with 1 mL of 0.1 M NaOH with 1% SDS (Sigma-Aldrich, St Louis, MO, USA). The obtained cell lysates were transmitted to scintillation vials and filled with 2 mL of scintillation fluid. Radioactivity was estimated by liquid scintillation counting on the Scintillation Counter 1900 TR, TRI-CARB (Packard, Perkin Elmer, Inc., San Jose, CA, USA). The intensity of DNA biosynthesis in the control cells was expressed in dpm of the radioactive thymidine incorporated in the DNA of the selected cell lines and was taken as 100%. Values from the tested compounds were expressed as a percentage of the control value.

### 4.5. Flow Cytometry Assessment of Annexin V Binding

Flow cytometry assessment was performed using the Annexin V binding Apoptosis Detection Kit II (BD Biosciences, San Diego, CA, USA) to examine the induction of apoptosis in DLD-1 and HT-29 colorectal adenocarcinoma cell lines. The procedure of detection was done in accordance with the manufacturer’s guidelines. The assay was performed as described in the literature. The tested compounds were added in 1.5 and 3 µM concentrations. The experiment was done at flow cytometer (BD FACSCanto II, Becton Dickinson Biosciences Systems, San Jose, CA, USA). Then, the obtained results were investigated with FACSDiva software (version 6.1.3, BD Biosciences Systems, San Jose, CA, USA) [51].

### 4.6. Acridine Orange/ethidium Bromide Fluorescent Staining

The method uses changes in the functioning of the cytoplasmic membrane undergoing apoptosis or necrosis. Modification in cell membrane permeability permits the penetration of fluorescent markers and specific staining of viable, dead, and apoptotic cells. DLD-1 and HT-29 cells were incubated with the tested compounds at 1.5 and 3 µM concentrations for 24 h. Cells were then stained with 10 µL of acridine orange and ethidium bromide incubated at room temperature for 5 min. Afterwards, the cells were examined in a fluorescence microscope Nikon Eclipse Ti connected to an inverted camera Nikon Instruments Inc., Melville, NY, USA) using 100× magnification. The results were parsed with NIS-Elements software (Nikon Instruments Inc., Melville, NY, USA) [52].

### 4.7. Analysis of Caspase-8 Enzymatic Activity

Caspase-8 activity was estimated with caspase-8: FLICA Caspase-8 Assay Kit (ImmunoChemistry Technologies, Bloomington, MN, USA). Both colon adenocarcinoma cell lines (DLD-1 and HT-29) were incubated for 24 h with the tested compounds at 1.5 and 3 µM. The test was performed as described in the literature. A flow cytometer (BD FACSCanto II, Becton Dickinson Biosciences Systems, San Jose, CA, USA) was used to measure the percent of activate caspase-8 in both cell lines. The data were collected and evaluated with FACSDiva software (BD Biosciences Systems, San Jose, CA, USA) [53].

### 4.8. Caspase-9 Enzymatic Activity Assay

Caspase-9 activity was measured using the FAM-FLICA Caspase-9 Kit (ImmunoChemistry Technologies, Bloomington, MN, USA) according to the manufacturer’s instructions. The DLD-1 and HT-29 cells were treated with the tested compounds at 1.5 and 3 µM concentrations for 24 h, and then harvested and washed with cold buffer PBS. Then 5 µL of diluted FLICA reagent and 2 µL of Hoechst 33342 were added to 93 µL of cell suspension and mixed by pipetting. The cells were incubated for 60 min at 37 °C. After incubation, the cells were washed twice with 400 µL apoptosis wash buffer and centrifuged at 300× *g*. After the last wash, the cells were resuspended in 100 µL apoptosis wash buffer and supplemented with 10 µg/mL PI. Analysis was performed using the BD FACSCanto II flow cytometer, and the results were analyzed with FACSDiva software (both from BD Biosciences Systems, San Jose, CA, USA) [53].

### 4.9. Caspase-3/7 Enzymatic Activity Assay

Caspase-3/7 was estimated with the FLICA Caspase-3/7 Assay Kit (ImmunoChemistry Technologies, Bloomington, MN, USA). Both DLD-1 and HT-29 cell lines were incubated for 24 h with the tested compounds and reference drugs at 1.5 and 3 µM. The test was performed as described in the literature [23]. The data were collected and evaluated with FACSDiva software (version 6.1.3, BD Biosciences Systems, San Jose, CA, USA).

### 4.10. Cell Cycle Analysis

Both DLD-1 and HT-29 colon cancer cell lines were exposed to the tested compounds **MM131** and 5-fluorouracil at concentrations 1.5 and 3.0 μM for 24 h. Afterwards the cells were collected (washed with PBS then treated with 0.05% trypsin/0.02% EDTA solution) and centrifuged (1000 rpm, 10 min, 4 °C). The supernatant was aspirated, and the cells were fixed in one milliliter of 70% ethanol and maintained overnight in a freezer at −20 °C. Prior to analysis, the ethanol was discarded, and the cells were resuspended in PBS with 50 μg/mL of DNase-free RNase A Solution (Promega, Madison, WI, USA), stained with PI (100 μg/mL of propidine iodide). The proportions of the cells in each phase of the cycle were measured by FACSCanto II flow cytometer (BD Biosciences Systems, San Jose, CA, USA) [49].

### 4.11. Determination of sICAM-1, mTOR, Cathepsin B, Beclin-1

High sensitivity assay kits (EIAab Science Co., Ltd, Wuhan, China) were used to determine the concentrations of proteins in the cell lysates after 24 h of incubation with the tested compounds at 0.5 and 1 µM concentration. Briefly, the trypsinized cells were washed three times with cold PBS and centrifuged at 1000× *g* for 5 min at 4 °C. The cells (1 × 10^6^) were suspended in a lysis buffer for whole cell lysates. After centrifugation, the supernatants were frozen immediately at −70 °C. The concentration of sICAM-1 in media, and mTOR, cathepsin B, Beclin-1 in cell lysates was measured. Cells without the addition of the compounds were treated as controls.

The microtiter plate provided in this kit had been pre-coated with an antibody specific to the analyzed antigen. The standards and samples were added to the appropriate microtiter plate wells. After two hours of incubation at 37 °C, the plate was incubated with biotin-conjugated antibody for one hour at 37 °C. Then, the microplate wells were aspirated and washed three times and then incubated with avidin conjugated to horseradish peroxidase (HRP). Then a TMB substrate solution was added to each well. The wells that contained the target antigen exhibited a change in color. The enzyme-substrate reaction was terminated by the addition of a sulfuric acid solution and the color change was measured spectrophotometrically at a wavelength of 450 ± 2 nm. concentrations in the samples were determined by comparing the O.D. of the samples to the standard curve [48].

## 5. Conclusions

**MM131** possesses promising anticancer properties. It inhibits the viability and proliferation of DLD-1 and HT-29 cells. The molecular mechanism of action is associated with the induction of both extrinsic and intrinsic apoptotic pathways. This is related to increased caspase-8 and caspase-9 activity. **MM131** leads to the inhibition of important proteins engaged in the progression and metastasis of colorectal cancer such as sICAM-1, cathepsin B, and mTOR.

## Data Availability

The datasets used and/or analyzed during the current study are available from the corresponding author on reasonable request.

## Supplementary Materials

The following are available online, ^1^H-NMR, ^13^C-NMR and HRMS spectra of compounds **2** and **MM131**.

## Sample Availability

Samples of the compounds **2** and **MM131** are available from the authors.

## Abbreviations

^13^C-NMR	Carbon-13 Nuclear Magnetic Resonance
^1^H-NMR	Hydrogen-1 Nuclear Magnetic Resonance
anti-EGFR	anti-epidermal growth factor receptor therapy
APAF1	apoptotic protease activating factor 1
ATCC	American Type Culture Collection
CK2	ubiquitous serine/threonine protein kinase
COX-2	cyclooxygenase-2
CRC	colorectal cancer
DLD-1	colorectal cancer cell line
DMSO	dimethyl sulfoxide
EDTA	ethylenediamine tetraacetic acid
EGFR	epidermal growth factor receptor
ELISA	Enzyme Immunosorbent Assay
ERK 1/2	extracellular signal-regulated kinase 1/2
EtOH	Ethanol
FADD	Fas-associated protein with death domain
FDA	Food and Drug Administration
FGFR	fibroblast growth factor receptor
GSK-3β	glycogen synthase kinase 3 β
H1299	non-small cell lung carcinoma cell line
HRP	horseradish peroxidase
HT-29	colorectal cancer cell line
IAP	inhibitors of apoptosis proteins
IC_50_	half maximal inhibitory concentration
JAK	janus-activated kinases
MAPK	mitogen-activated protein kinase
MDA-MB-435	melanoma cancer cell line
MeCN	acetonitrile
MMP-2	matrix metalloproteinase 2
MMP-9	matrix metalloproteinase 9
mTOR	mechanistic target of rapamycin
MTT	3-(4,5-dimethylthiazol-2-yl)-2,5-diphenyltetrazolium bromide
NaN_3_	Sodium azide
PBS	Phosphate Buffered Saline
PI	Propidium Iodide
PI3K	phosphoinositide 3-kinase
Pim-1	proviral integration site for Moloney murine leukemia virus-1 kinase
rt	room temperature
SD	Standard Deviation
SDS	Sodium Dodecyl Sulfate
SEM	standard error of the mean
sICAM-1	soluble intercellular adhesion molecule-1
SYK	spleen tyrosine kinase
T-47D	breast cancer cell line
TCA	Trichloroacetic acid
Tie-2	tyrosine kinase with immunoglobulin and epidermal growth factorhomology domains-2
TLC	thin layer chromatography
TMB	Tetramethylbenzidine
TMS	tetramethylsilane
TNF	tumor necrosis factor
TRAIL	tumor necrosis factor (TNF)-related apoptosis-inducing ligand
VEGF	vascular endothelial growth factor
VEGFR	vascular endothelial growth factor receptor
