# Supplementary material for: The Anticancer Action of a Novel 1,2,4-Triazine Sulfonamide Derivative in Colon Cancer Cells
Source: Molecules. 2021 Apr 2;26(7):2045. doi: 10.3390/molecules26072045 (PMC8038278; doi:10.3390/molecules26072045)

Supplementary Materials

The Anticancer Action of a Novel 1,2,4-Triazine Sulfonamide Derivative in Colon Cancer Cells

Agnieszka Gornowicz [1]*, Anna Szymanowska [1], Mariusz Mojzych [2], Robert Czarnomysy [3], Krzysztof Bielawski [3] and Anna Bielawska [1]

[1] Department of Biotechnology, Medical University of Bialystok, 15-222 Bialystok, Poland; agnieszka.gornowicz@umb.edu.pl (A.G.); anna.szymanowska@umb.edu.pl (A.S.); anna.bielawska@umb.edu.pl (A.B.)
[2] Department of Chemistry, Siedlce University of Natural Sciences and Humanities, 08-110 Siedlce, Poland; mmojzych@yahoo.com (M.M.)
[3] Department of Synthesis and Technology of Drugs, Medical University of Bialystok, 15-222 Bialystok, Poland; robert.czarnomysy@umb.edu.pl (R.C.); kbiel@umb.edu.pl (K.B.)
* Correspondence: agnieszka.gornowicz@umb.edu.pl (A.G)

Compound **2**

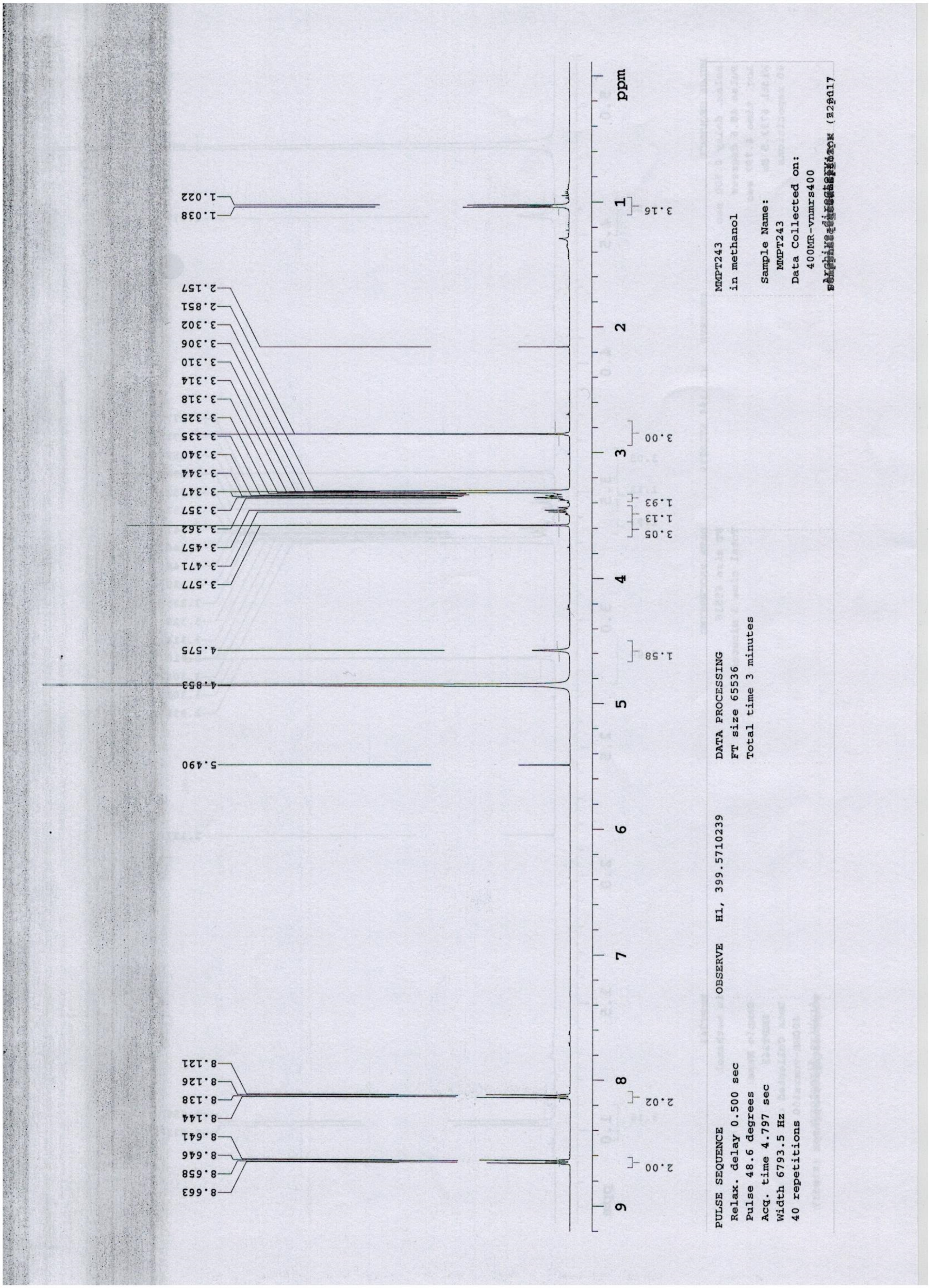
PULSE SEQUENCE
Relax. delay 0.500 sec
Pulse 48.6 degrees
Acq. time 4.797 sec
Width 6793.5 Hz
40 repetitions
OBSERVE H1, 399.5710239
DATA PROCESSING
FT size 65536
Total time 3 minutes
MMPT243
in methanol
Sample Name:
MMPT243
Data Collected on:
400MR-vnmrs400
ppm

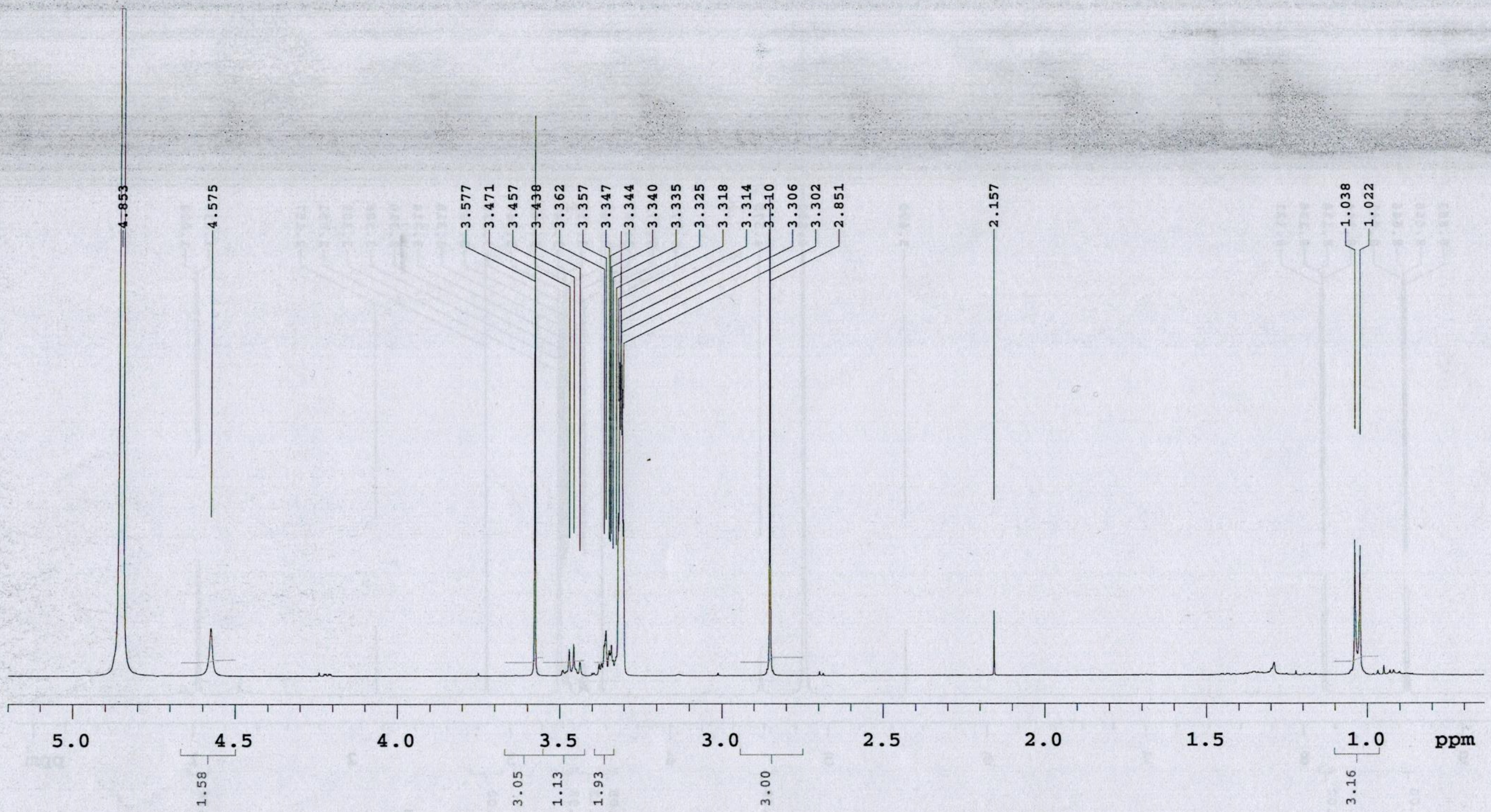

PULSE SEQUENCE
Relax. delay 0.500 sec
Pulse 48.6 degrees
Acq. time 4.797 sec
Width 6793.5 Hz
40 repetitions

OBSERVE H1, 399.5710239

DATA PROCESSING
FT size 65536
Total time 3 minutes

MMPT243
in methanol

Sample Name:
MMPT243
Data Collected on:
400MR-vnmrs400
[illegible]

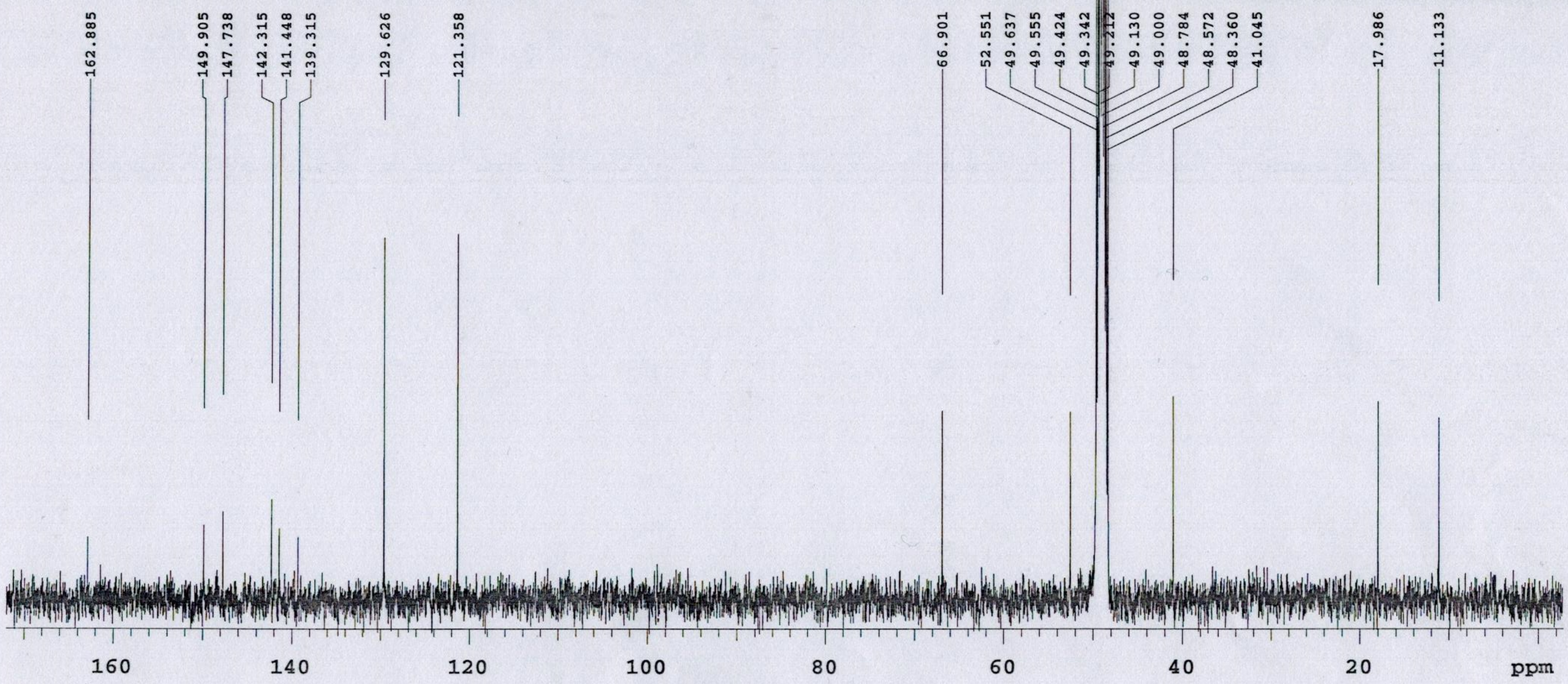

PULSE SEQUENCE
Relax. delay 1.500 sec
Pulse 38.5 degrees
Acq. time 2.674 sec
Width 24509.8 Hz
14400 repetitions

OBSERVE C13, 100.4720710
DECOUPLE H1, 399.5730276
Power 37 dB
continuously on
WALTZ-16 modulated

DATA PROCESSING
Line broadening 1.5 Hz
FT size 131072
Total time 16.7 hours

MMPT243
in methanol

Sample Name:
MMPT243
Data Collected on:
[illegible]

Compound **MM131**

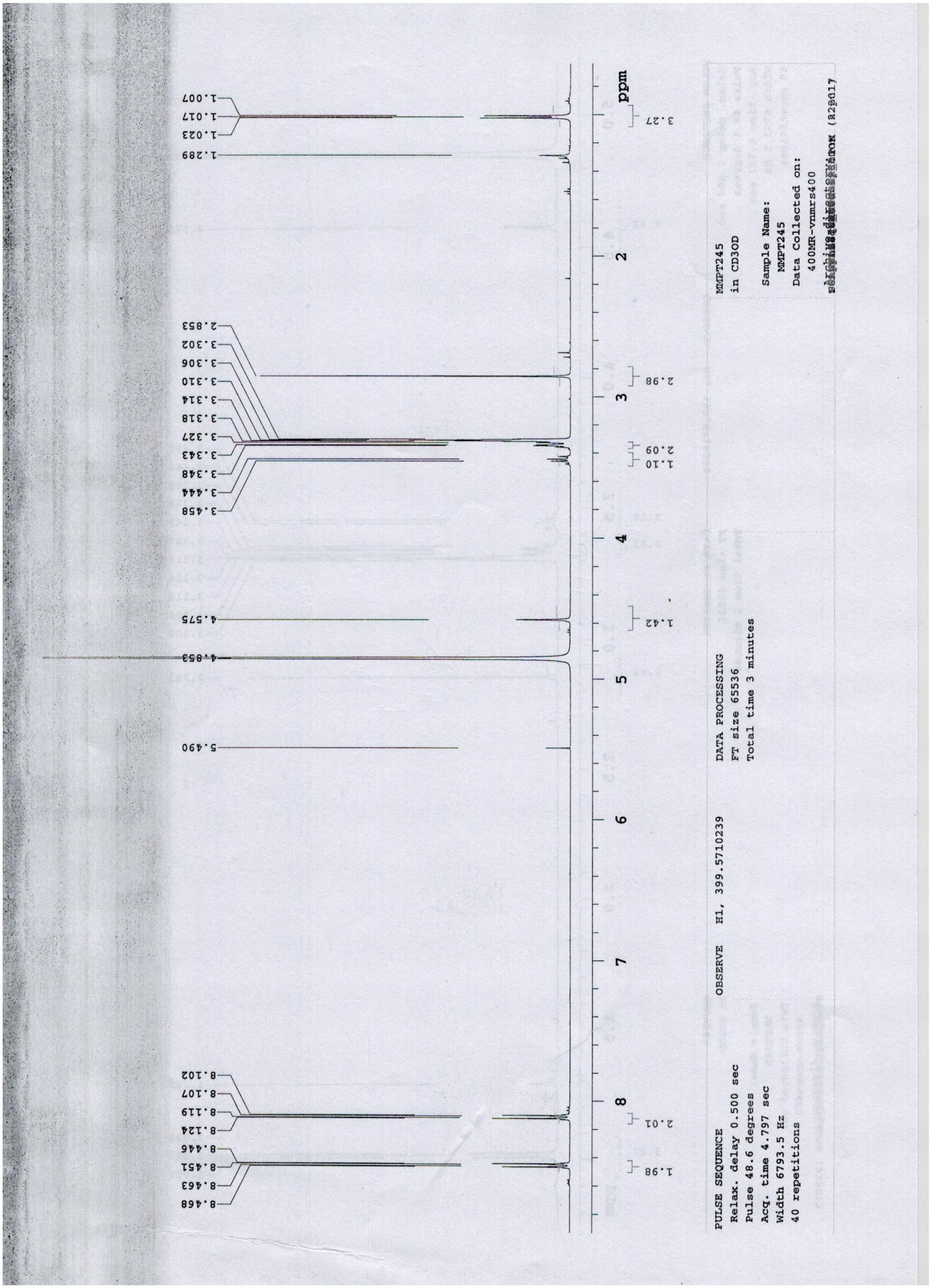

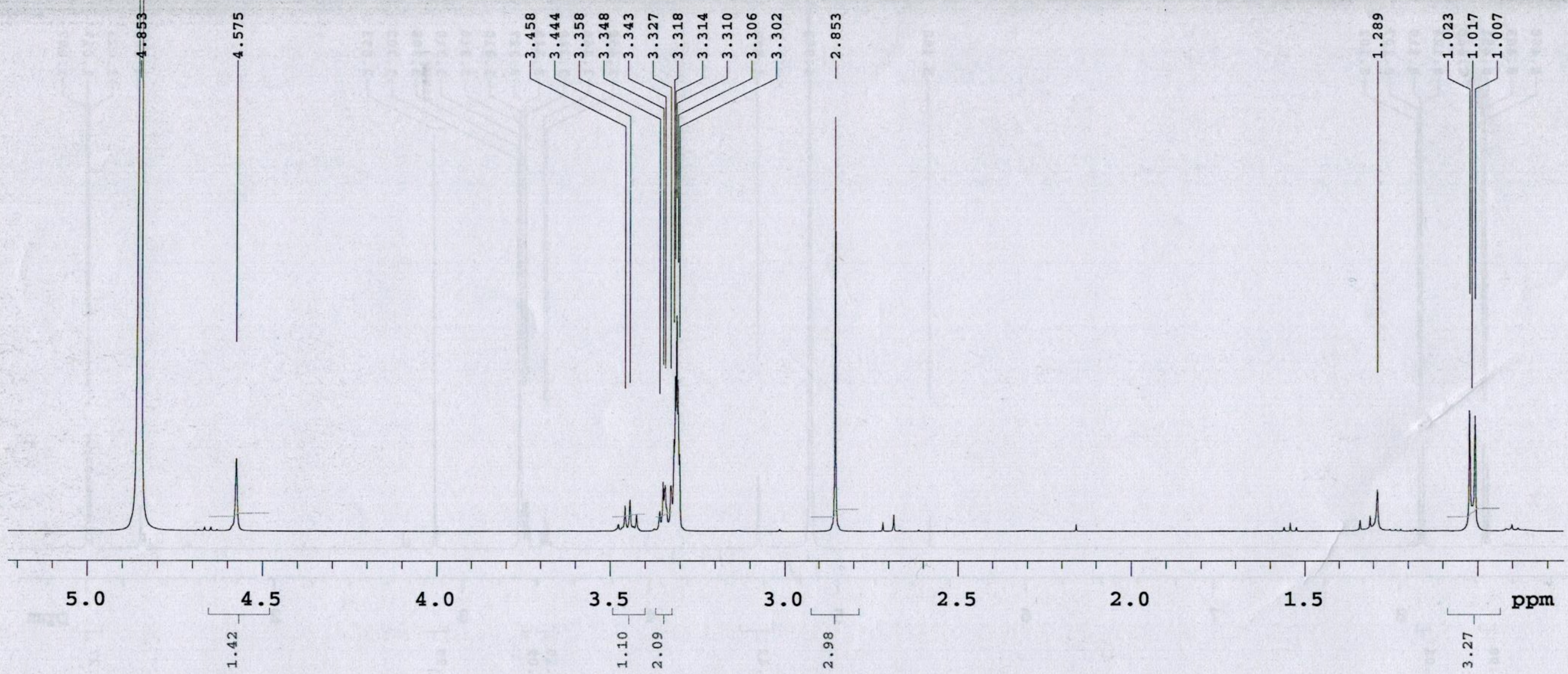

PULSE SEQUENCE
Relax. delay 0.500 sec
Pulse 48.6 degrees
Acq. time 4.797 sec
Width 6793.5 Hz
40 repetitions

OBSERVE H1, 399.5710239

DATA PROCESSING
FT size 65536
Total time 3 minutes

MMPT245
in CD3OD

Sample Name:
MMPT245
Data Collected on:
400MR-vnmrs400
Archive directory: [illegible]

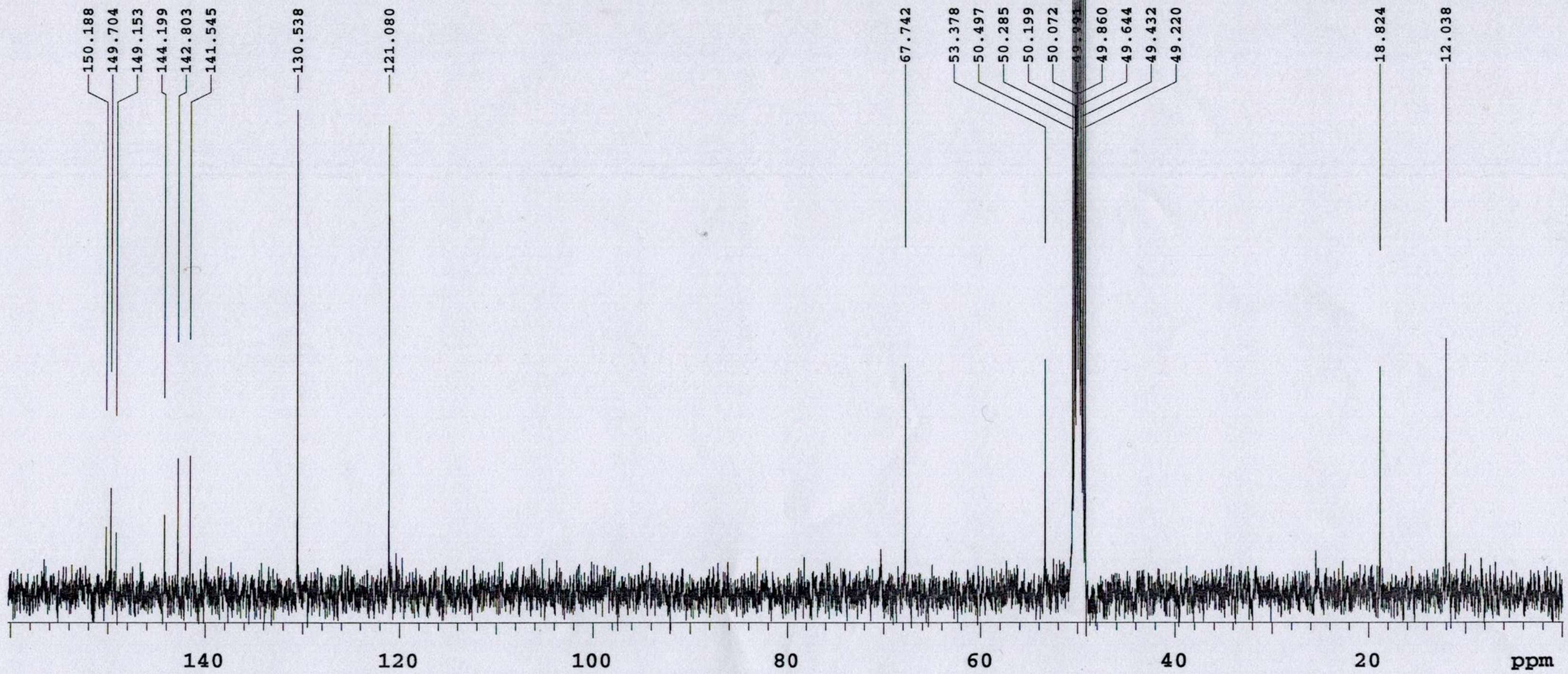

PULSE SEQUENCE
Relax. delay 1.500 sec
Pulse 38.5 degrees
Acq. time 2.674 sec
Width 24509.8 Hz
1984 repetitions

OBSERVE C13, 100.4719853
DECOUPLE H1, 399.5730276
Power 37 dB
continuously on
WALTZ-16 modulated

DATA PROCESSING
Line broadening 1.5 Hz
FT size 131072
Total time 2.3 hours

MMPT245
in CD3OD

Sample Name:
MMPT245
Data Collected on:
400MR-vnmrs400
[illegible]

Compound **2**

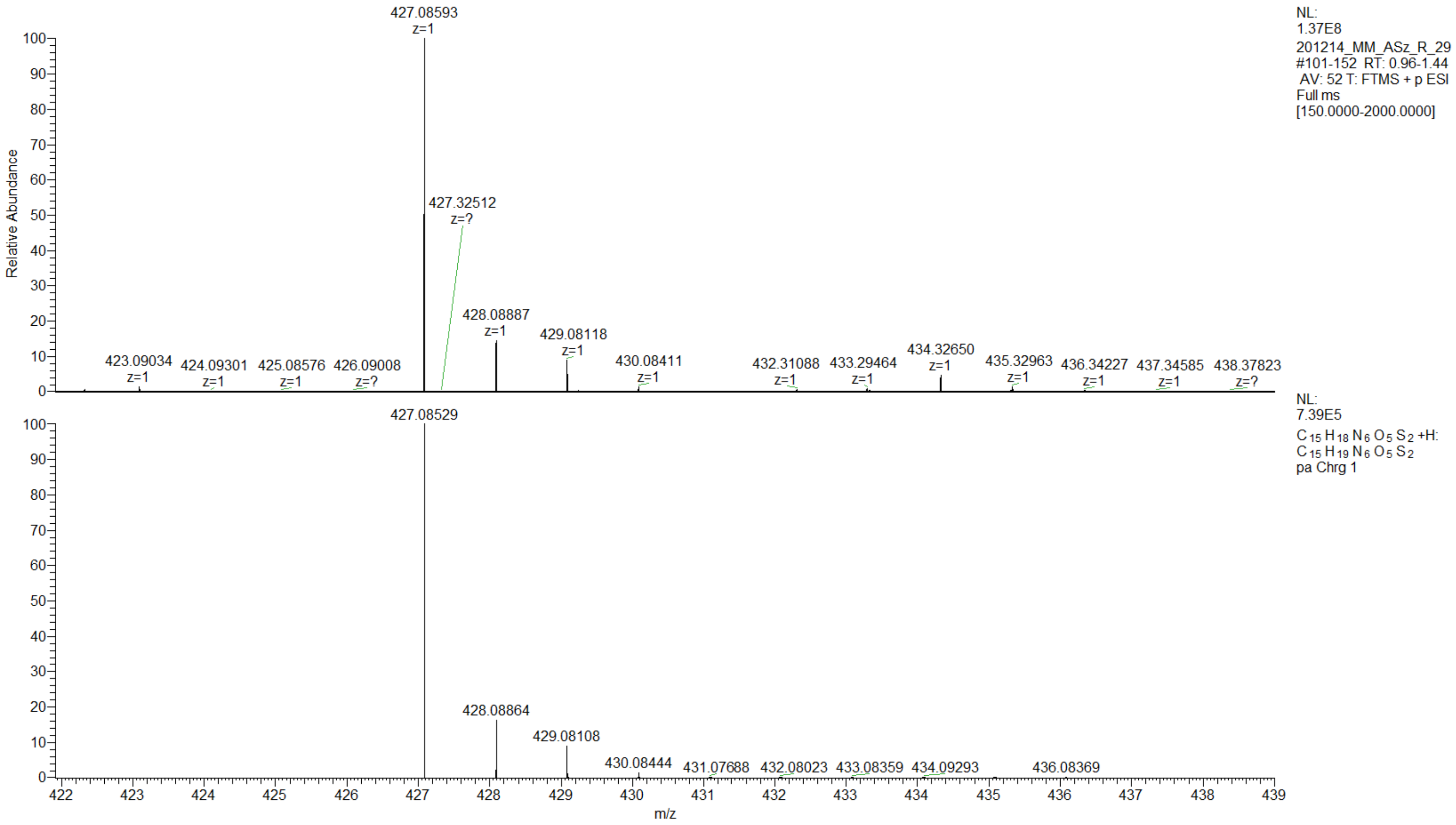

427.08593
z=1
427.32512
z=?
428.08887
z=1
429.08118
z=1
423.09034
z=1
424.09301
z=1
425.08576
z=1
426.09008
z=?
430.08411
z=1
432.31088
z=1
433.29464
z=1
434.32650
z=1
435.32963
z=1
436.34227
z=1
437.34585
z=1
438.37823
z=?
NL:
1.37E8
201214_MM_ASz_R_29
#101-152 RT: 0.96-1.44
AV: 52 T: FTMS + p ESI
Full ms
[150.0000-2000.0000]
Relative Abundance
427.08529
428.08864
429.08108
430.08444
431.07688
432.08023
433.08359
434.09293
436.08369
NL:
7.39E5
C 15 H 18 N 6 O 5 S 2 +H:
C 15 H 19 N 6 O 5 S 2
pa Chrg 1
m/z

Compound **MM131**

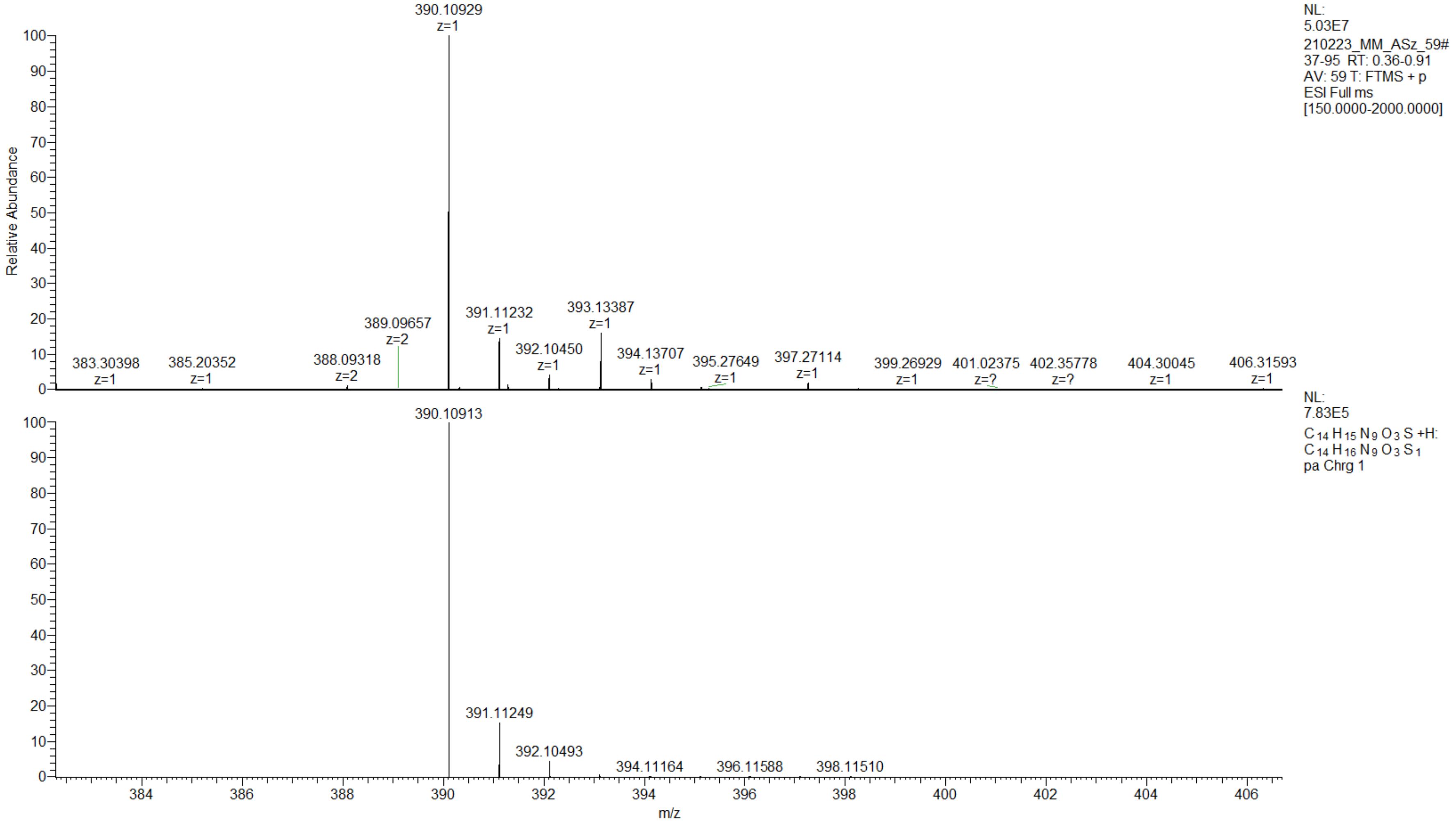

Supplement: Supplementary file 1 [file molecules-26-02045-s001.pdf]
